# Report of Deaths in Buffalo for the Month of May, 1855

**Published:** 1855-07

**Authors:** J. Root

**Affiliations:** Health Physician


### ART. V.—*Report of Deaths in Buffalo for the month*

| DISEASES. | Males. | Females. | Total. | AGE. Under 1 year. Male. | AGE. Under 1 year. Female. | AGE. 1 to 2 years. Male. | AGE. 1 to 2 years. Female. |
|---|---|---|---|---|---|---|---|
| Apoplexy, | 2 | | 2 | | | | |
| Consumption, | 10 | 14 | 24 | | 1 | | |
| Convulsions, | 3 | 5 | 8 | 1 | 5 | 1 | |
| Cyanosis, | | 1 | 1 | | | | |
| Croup, | 4 | 2 | 6 | 2 | 1 | 1 | |
| Chronic Bronchitis, | 1 | | 1 | | | | |
| Cancer of Stomach, | 1 | | 1 | | | | |
| Debility, | 5 | 3 | 8 | 4 | 2 | | |
| Defective Development, | 1 | | 1 | 1 | | | |
| Dropsy, | 1 | 2 | 3 | | | | |
| " of Brain, | 1 | 2 | 3 | 1 | | | 1 |
| Drowned, | 5 | 1 | 6 | | | | |
| Dentition, | 1 | 2 | 3 | | 2 | 1 | |
| Diarrhœa, | | 2 | 2 | | 2 | | |
| Erysipelas, | 1 | | 1 | | | | |
| Epilepsy, | 1 | | 1 | | | | |
| Fever, Typhoid, | 2 | 1 | 3 | | | | |
| " Scarlet, | 2 | 2 | 4 | | | | 1 |
| " Remittent, | | 1 | 1 | | | | |
| " Intermittent, | 1 | | 1 | | | | |
| " Puerperal, | 1 | | 1 | | | | |
| Gastro-enteritis, | 1 | | 1 | | | 1 | |
| Hydrocephalus, | 3 | 2 | 5 | 2 | 1 | | 1 |
| Inflammation of Brain, | | 1 | 1 | | 1 | | |
| " " Bowels, | | 1 | 1 | | | | |
| Inanition, | | 1 | 1 | | | | |
| Old Age, | 3 | 1 | 4 | | | | |
| Pneumonia, | 1 | 1 | 2 | | | 1 | |
| Pleurisy, | | 1 | 1 | | | | |
| Pulmonary Apoplexia, | 1 | | 1 | | | 1 | |
| Paralysis, | 1 | 1 | 2 | | | | |
| Peritonitis, | 1 | 1 | 2 | | | | |
| Scalded, | 1 | | 1 | | | 1 | |
| Suicide, | | 1 | 1 | | | | |
| Scrofula, | | 1 | 1 | | | | |
| Still-born, | 3 | 1 | 4 | 3 | 1 | | |
| Typhoid Pneumonia, | 1 | | 1 | | | | |
| Unknown, | 7 | 5 | 12 | 5 | 5 | | |
| Uterine Hæmorrhage, | | 1 | 1 | | | | |
| Worms, | 2 | | 2 | 1 | | | |
| Whooping Cough, | 1 | 4 | 5 | | 2 | | 2 |
| Totals, | 69 | 61 | 130 | 20 | 23 | 7 | 5 |

NATIVITIES.—American, 55. German, 29. English, 2. Irish, 16. Scotch, 1. Swiss, 1.

*of May*, 1855. By J. Root, M. D., Health Physician.

AGE.

| 2—5 | | 5—10 | | 10—15 | | 15—20 | | 20—25 | | 25—30 | | 30—40 | | 40—50 | | 50—60 | | 60—70 | | 70—80 | | 80—90 | | 90—100 | | 100 and upward. | | Not given. | |
|---|---|---|---|---|---|---|---|---|---|---|---|---|---|---|---|---|---|---|---|---|---|---|---|---|---|---|---|---|---|---|---|
| Male. | Female. | Male. | Female. | Male. | Female. | Male. | Female. | Male. | Female. | Male. | Female. | Male. | Female. | Male. | Female. | Male. | Female. | Male. | Female. | Male. | Female. | Male. | Female. | Male. | Female. | Male. | Female. | Male. | Female. | Male. | Female. |
| .. | .. | .. | .. | .. | .. | .. | .. | .. | .. | .. | .. | .. | .. | 1 | .. | .. | .. | .. | .. | 1 | .. | .. | .. | .. | .. | .. | .. | .. | .. | .. | .. |
| 1 | 1 | .. | .. | 2 | 1 | .. | .. | 2 | .. | 1 | 2 | 2 | 6 | 2 | 1 | .. | .. | .. | 2 | .. | .. | .. | .. | .. | .. | .. | .. | .. | .. | .. | .. |
| 1 | .. | .. | .. | .. | .. | .. | .. | .. | .. | .. | .. | .. | .. | .. | .. | .. | .. | .. | .. | .. | .. | .. | .. | .. | .. | .. | .. | .. | .. | .. | .. |
| .. | 1 | .. | .. | .. | .. | .. | .. | .. | .. | .. | .. | .. | .. | .. | .. | .. | .. | .. | .. | .. | .. | .. | .. | .. | .. | .. | .. | .. | .. | .. | .. |
| 1 | .. | .. | 1 | .. | .. | .. | .. | .. | .. | .. | .. | .. | .. | .. | .. | .. | .. | .. | .. | .. | .. | .. | .. | .. | .. | .. | .. | .. | .. | .. | .. |
| .. | .. | .. | .. | .. | .. | .. | .. | .. | .. | .. | .. | .. | .. | .. | .. | .. | .. | .. | .. | 1 | .. | .. | .. | .. | .. | .. | .. | .. | .. | .. | .. |
| .. | .. | .. | .. | .. | .. | .. | .. | .. | .. | .. | .. | .. | .. | 1 | .. | .. | .. | .. | .. | .. | .. | .. | .. | .. | .. | .. | .. | .. | .. | .. | .. |
| .. | .. | 1 | .. | .. | .. | .. | .. | .. | .. | .. | .. | .. | 1 | .. | .. | .. | .. | .. | .. | .. | .. | .. | .. | .. | .. | .. | .. | .. | .. | .. | .. |
| .. | .. | .. | .. | .. | .. | .. | .. | .. | .. | .. | .. | .. | .. | .. | .. | .. | .. | .. | .. | .. | .. | .. | .. | .. | .. | .. | .. | .. | .. | .. | .. |
| .. | .. | .. | .. | .. | .. | .. | .. | .. | .. | .. | .. | 1 | .. | .. | .. | .. | 1 | .. | .. | .. | .. | .. | 1 | .. | .. | .. | .. | .. | .. | .. | .. |
| .. | .. | .. | .. | .. | .. | .. | 1 | .. | .. | .. | .. | .. | .. | .. | .. | .. | .. | .. | .. | .. | .. | .. | .. | .. | .. | .. | .. | .. | .. | .. | .. |
| 2 | .. | .. | 1 | .. | .. | .. | .. | 1 | .. | .. | .. | 1 | .. | 1 | .. | .. | .. | .. | .. | .. | .. | .. | .. | .. | .. | .. | .. | .. | .. | .. | .. |
| .. | .. | .. | .. | .. | .. | .. | .. | .. | .. | .. | .. | .. | .. | .. | .. | .. | .. | .. | .. | .. | .. | .. | .. | .. | .. | .. | .. | .. | .. | .. | .. |
| .. | .. | .. | .. | .. | .. | .. | .. | .. | .. | .. | .. | .. | .. | .. | .. | .. | .. | .. | .. | .. | .. | .. | .. | .. | .. | .. | .. | .. | .. | .. | .. |
| .. | .. | .. | .. | .. | .. | .. | 1 | .. | .. | .. | .. | .. | .. | .. | .. | .. | .. | .. | .. | .. | .. | .. | .. | .. | .. | .. | .. | .. | .. | .. | .. |
| .. | .. | .. | .. | .. | .. | .. | .. | .. | .. | .. | .. | .. | 1 | .. | .. | .. | .. | .. | .. | .. | .. | .. | .. | .. | .. | .. | .. | .. | .. | .. | .. |
| .. | .. | .. | .. | 1 | .. | .. | .. | 1 | .. | .. | .. | .. | .. | .. | .. | .. | 1 | .. | .. | .. | .. | .. | .. | .. | .. | .. | .. | .. | .. | .. | .. |
| .. | 1 | 2 | .. | .. | .. | .. | .. | .. | .. | .. | .. | .. | .. | .. | .. | .. | .. | .. | .. | .. | .. | .. | .. | .. | .. | .. | .. | .. | .. | .. | .. |
| .. | .. | .. | .. | .. | .. | .. | .. | .. | .. | .. | .. | .. | .. | .. | .. | .. | .. | .. | .. | .. | .. | .. | 1 | .. | .. | .. | .. | .. | .. | .. | .. |
| .. | .. | .. | .. | .. | .. | .. | .. | .. | .. | .. | .. | 1 | .. | .. | .. | .. | .. | .. | .. | .. | .. | .. | .. | .. | .. | .. | .. | .. | .. | .. | .. |
| .. | .. | .. | .. | .. | .. | .. | .. | .. | 1 | .. | .. | .. | .. | .. | .. | .. | .. | .. | .. | .. | .. | .. | .. | .. | .. | .. | .. | .. | .. | .. | .. |
| .. | .. | .. | .. | .. | .. | .. | .. | .. | .. | .. | .. | .. | .. | .. | .. | .. | .. | .. | .. | .. | .. | .. | .. | .. | .. | .. | .. | .. | .. | .. | .. |
| .. | .. | 1 | .. | .. | .. | .. | .. | .. | .. | .. | .. | .. | .. | .. | .. | .. | .. | .. | .. | .. | .. | .. | .. | .. | .. | .. | .. | .. | .. | .. | .. |
| .. | .. | .. | .. | .. | .. | .. | .. | .. | .. | .. | .. | .. | .. | .. | .. | .. | .. | .. | .. | .. | .. | .. | .. | .. | .. | .. | .. | .. | .. | .. | .. |
| .. | .. | .. | .. | .. | .. | .. | .. | .. | .. | .. | .. | .. | .. | .. | .. | .. | .. | .. | 1 | .. | .. | .. | .. | .. | .. | .. | .. | .. | .. | .. | .. |
| .. | .. | .. | 1 | .. | .. | .. | .. | .. | .. | .. | .. | .. | .. | .. | .. | .. | .. | .. | .. | .. | .. | .. | .. | .. | .. | .. | .. | .. | .. | .. | .. |
| .. | .. | .. | .. | .. | .. | .. | .. | .. | .. | .. | .. | .. | .. | .. | .. | .. | .. | 3 | 1 | .. | .. | .. | .. | .. | .. | .. | .. | .. | .. | .. | .. |
| .. | .. | .. | .. | .. | .. | .. | .. | .. | .. | .. | .. | .. | 1 | .. | .. | .. | .. | .. | .. | .. | .. | .. | .. | .. | .. | .. | .. | .. | .. | .. | .. |
| .. | .. | .. | .. | .. | 1 | .. | .. | .. | .. | .. | .. | .. | .. | .. | .. | .. | .. | .. | .. | .. | .. | .. | .. | .. | .. | .. | .. | .. | .. | .. | .. |
| .. | .. | .. | .. | .. | .. | .. | .. | .. | .. | .. | .. | .. | .. | .. | .. | .. | .. | .. | .. | .. | .. | .. | .. | .. | .. | .. | .. | .. | .. | .. | .. |
| .. | .. | .. | 1 | .. | .. | .. | .. | .. | .. | .. | .. | .. | .. | .. | .. | .. | .. | .. | .. | .. | .. | 1 | .. | .. | .. | .. | .. | .. | .. | .. | .. |
| .. | .. | .. | .. | .. | .. | .. | .. | 1 | .. | .. | .. | .. | .. | .. | .. | .. | .. | .. | .. | .. | 1 | .. | .. | .. | .. | .. | .. | .. | .. | .. | .. |
| .. | .. | .. | .. | .. | .. | .. | .. | .. | .. | .. | .. | .. | .. | .. | .. | .. | .. | .. | .. | .. | .. | .. | .. | .. | .. | .. | .. | .. | .. | .. | .. |
| .. | .. | .. | .. | .. | .. | .. | .. | .. | .. | .. | .. | .. | 1 | .. | .. | .. | .. | .. | .. | .. | .. | .. | .. | .. | .. | .. | .. | .. | .. | .. | .. |
| .. | 1 | .. | .. | .. | .. | .. | .. | .. | .. | .. | .. | .. | .. | .. | .. | .. | .. | .. | .. | .. | .. | .. | .. | .. | .. | .. | .. | .. | .. | .. | .. |
| .. | .. | .. | .. | .. | .. | .. | .. | .. | .. | .. | .. | .. | .. | .. | .. | .. | .. | .. | .. | .. | .. | .. | .. | .. | .. | .. | .. | .. | .. | .. | .. |
| .. | .. | .. | .. | .. | .. | .. | .. | 1 | .. | .. | .. | .. | .. | .. | .. | .. | .. | .. | .. | .. | .. | .. | .. | .. | .. | .. | .. | .. | .. | .. | .. |
| 1 | .. | .. | .. | .. | .. | .. | .. | .. | .. | .. | .. | .. | .. | .. | .. | 1 | .. | .. | .. | .. | .. | .. | .. | .. | .. | .. | .. | .. | .. | .. | .. |
| .. | .. | .. | .. | .. | .. | .. | .. | .. | .. | .. | .. | .. | 1 | .. | .. | .. | .. | .. | .. | .. | .. | .. | .. | .. | .. | .. | .. | .. | .. | .. | .. |
| 1 | .. | .. | .. | .. | .. | .. | .. | .. | .. | .. | .. | .. | .. | .. | .. | .. | .. | .. | .. | .. | .. | .. | .. | .. | .. | .. | .. | .. | .. | .. | .. |
| 1 | .. | .. | .. | .. | .. | .. | .. | .. | .. | .. | .. | .. | .. | .. | .. | .. | .. | .. | .. | .. | .. | .. | .. | .. | .. | .. | .. | .. | .. | .. | .. |
| .. | .. | .. | .. | .. | .. | .. | .. | .. | .. | .. | .. | .. | .. | .. | .. | .. | .. | .. | .. | .. | .. | .. | .. | .. | .. | .. | .. | .. | .. | .. | .. |
| .. | .. | .. | .. | .. | .. | .. | .. | .. | .. | .. | .. | .. | .. | .. | .. | .. | .. | .. | .. | .. | .. | .. | .. | .. | .. | .. | .. | .. | .. | .. | .. |
| .. | .. | .. | .. | .. | .. | .. | .. | .. | .. | .. | .. | .. | .. | .. | .. | .. | .. | .. | .. | .. | .. | .. | .. | .. | .. | .. | .. | .. | .. | .. | .. |
| .. | .. | .. | .. | .. | .. | .. | .. | .. | .. | .. | .. | .. | .. | .. | .. | .. | .. | .. | .. | .. | .. | .. | .. | .. | .. | .. | .. | .. | .. | .. | .. |
| .. | .. | .. | .. | .. | .. | .. | .. | .. | .. | .. | .. | .. | .. | .. | .. | .. | .. | .. | .. | .. | .. | .. | .. | .. | .. | .. | .. | .. | .. | .. | .. |
| .. | .. | .. | .. | .. | .. | .. | .. | .. | .. | .. | .. | .. | .. | .. | .. | .. | .. | .. | .. | .. | .. | .. | .. | .. | .. | .. | .. | .. | .. | .. | .. |
| .. | .. | .. | .. | .. | .. | .. | .. | .. | .. | .. | .. | .. | .. | .. | .. | .. | .. | .. | .. | .. | .. | .. | .. | .. | .. | .. | .. | .. | .. | .. | .. |
| .. | .. | .. | .. | .. | .. | .. | .. | .. | .. | .. | .. | .. | .. | .. | .. | .. | .. | .. | .. | .. | .. | .. | .. | .. | .. | .. | .. | .. | .. | .. | .. |
| .. | .. | .. | .. | .. | .. | .. | .. | .. | .. | .. | .. | .. | .. | .. | .. | .. | .. | .. | .. | .. | .. | .. | .. | .. | .. | .. | .. | .. | .. | .. | .. |
| .. | .. | .. | .. | .. | .. | .. | .. | .. | .. | .. | .. | .. | .. | .. | .. | .. | .. | .. | .. | .. | .. | .. | .. | .. | .. | .. | .. | .. | .. | .. | .. |
| 8 | 4 | 4 | 4 | 3 | 2 | 0 | 2 | 6 | 1 | 1 | 2 | 5 | 11 | 5 | 1 | 1 | 2 | 3 | 4 | 2 | 1 | 1 | 2 | 0 | 0 | 0 | 0 | 0 | 0 | 0 | 0 |

French, 2. Canadian, 2. Hollander, 1. Colored, 1. Not given, 20.—Total, 130.

